# Investigating the Therapeutic Potential of Crude Leech Saliva Based on Its Anticancer, Antioxidant, and Anti-Inflammatory Effects

**DOI:** 10.3390/cimb47050328

**Published:** 2025-05-03

**Authors:** Alican Bilden, İlhan Sabancılar, Serap Yalçın Azarkan, Kenan Karadağlı, Seçkin Kaya, Merve Kahraman, Muttalip Çiçek

**Affiliations:** 1 Department of Medical Parasitology, Faculty of Medicine, Kırşehir Ahi Evran University, Kırşehir 40100, Turkey; merve.kahraman@ahievran.edu.tr (M.K.); muttalipcicek@ahievran.edu.tr (M.Ç.); 2 Medical Services and Techniques Department, Bitlis Eren University, Bitlis 13100, Turkey; ilhansabancilar@hotmail.com; 3 Department of Medical Pharmacology, Faculty of Medicine, Kırşehir Ahi Evran University, Kırşehir 40100, Turkey; syalcin@ahievran.edu.tr; 4 Department of Medical Parasitology, Faculty of Medicine, Siirt Education and Research Hospital, Siirt 56100, Turkey; k.kenan1876@gmail.com; 5 Department of Medical Biochemistry, Institute of Health Sciences, Ataturk University, Erzurum 25240, Turkey; seckin.kaya24@ogr.atauni.edu.tr

**Keywords:** *Hirudo verbana*, HUVEC, OVCAR3, cell viability, antioxidant activity, cytokines

## Abstract

Leech therapy is a biotherapeutic approach that has been traditionally used for centuries and is currently being re-evaluated in modern medicine. The efficacy of this treatment is attributed to various bioactive compounds found in leech saliva, which exhibit anticoagulant, anti-inflammatory, antioxidant, and anticancer properties. It has been demonstrated that leech saliva possesses the potential to modulate inflammatory processes and apoptotic mechanisms. In this study, the therapeutic potential of the saliva of *Hirudo verbana* was evaluated, and its biological and pharmacological effects were comprehensively investigated. The anticancer effects, antioxidant capacity, and anti-inflammatory activity of the crude leech saliva were assessed using human umbilical vein endothelial cells and epithelial ovarian cancer cells. The chemical composition of the saliva was analyzed using gas chromatography–mass spectrometry, while the protein content was determined by the Bradford assay. Antioxidant activity was measured using the 2,2-diphenyl-1-picrylhydrazyl radical scavenging assay, inflammatory effects were evaluated by Enzyme-Linked ImmunoSorbent Assay, and cell viability was determined using the 3-(4,5-dimethylthiazol-2-yl)-2,5-diphenyltetrazolium bromide assay. The findings revealed that crude leech saliva had a minimal effect on healthy cells but showed a selective effect on the viability of ovarian cancer cells. At low concentrations (3.13%), 99.16% of healthy cells remained viable, whereas this rate decreased to 89.25% in cancer cells; at high concentrations (50%), cell viability in cancer cells declined to 63.02%. Gas chromatography–mass spectrometry analysis identified compounds such as gibberellic acid and 6-[(4-methoxyphenyl)methoxy]-4,4,5,7,8-pentamethyl-3H-chromen-2-one, which demonstrated high affinity for the antiapoptotic proteins Bcl-2 and Survivin in molecular docking analyses. In conclusion, the crude leech saliva was confirmed to possess anti-inflammatory, antioxidant, and anticancer properties. However, further biochemical and clinical research is needed to elucidate the underlying mechanisms of these biological effects in greater detail.

## 1. Introduction

Traditional and complementary medicine practices have been used for centuries across various cultures to treat a wide range of diseases. One such practice is hirudotherapy (leech therapy), which represents a rare example of invertebrate use in medical treatment. Leech therapy has been known since ancient times and has been applied both in human and veterinary medicine [1]. The earliest records of hirudotherapy date back to around 1500 BC in Egypt [2]. The renowned Roman physician Galen (AD 130–201) provided detailed descriptions of the diseases treated with leeches, their application methods, removal techniques, and management of excessive bleeding [3]. Likewise, Avicenna (Ibn Sina, AD 980–1037) mentioned the purposes and indications of medicinal leeches in his medical encyclopedia *The Canon of Medicine*. Today, leech therapy has regained importance as a supportive modality in modern medicine, especially in plastic and reconstructive surgery [4]. Furthermore, in 2004, the U.S. Food and Drug Administration (FDA) approved the use of leeches for relieving venous congestion in plastic and microsurgery procedures (FDA, K040062) [5].

Cancer continues to be a major global health concern, leading to substantial mortality despite the availability of current treatment options. According to recent statistics, annual deaths attributed to breast, gastric, liver, lung, and colorectal cancers are approximately 685,000; 769,000; 830,000; 1.8 million; and 916,000, respectively [6]. Ovarian cancer also ranks among the most lethal malignancies worldwide [7]. In addition to cancer, chronic and autoimmune diseases remain leading causes of death. Oxidative stress and inflammation are central contributors to the pathogenesis of these disorders. Oxidative stress, induced by excessive free radical production, significantly disrupts cellular structures and functions. This process can lead to lipid peroxidation, DNA damage, and accelerated cell death. Over time, such damage impairs immune cell function, laying the groundwork for chronic illnesses such as cardiovascular diseases, cancer, inflammatory disorders, and neuropathies. These processes can also reduce treatment efficacy and complicate recovery [8,9]. Consequently, researchers are increasingly focused on strengthening existing therapies and exploring alternative strategies [10]—hirudotherapy being one of them.

Leech saliva (LS) contains numerous biologically and pharmacologically active bioactive compounds [11]. These substances can elicit various biological effects upon application to the body, including anticoagulant, antioxidant, anticancer, fibrinolytic, anti-inflammatory, analgesic, vasodilatory, antimicrobial, viscosity-enhancing, and enzyme-inhibitory activities [12]. Several studies have demonstrated the presence of antimetastatic and anticancer agents in LS, particularly in the context of different cancer types and metastasis management [13]. For instance, one study revealed that LS significantly inhibited the growth of MCF-7 breast cancer cells in vitro [14]. In another study, leech saliva extract (LSE) was shown to significantly reduce tumor volume and serum PSA levels in prostate cancer models. Furthermore, it inhibited angiogenesis and cell adhesion while enhancing cleaved caspase-3 expression, thereby promoting apoptosis [15]. Additionally, LS has been found to suppress the VEGFA signaling pathway, exerting a strong anti-angiogenic effect that is further enhanced when incorporated into liposomal delivery systems [16].

Beyond its anticancer effects, LS has also demonstrated strong antioxidant and anti-inflammatory potential. Several studies have examined its impact on inflammation and oxidative stress, suggesting that compounds such as endocannabinoids and lipids may modulate inflammatory pathways and mitigate oxidative damage [17]. Systematic reviews have further shown that LS is clinically effective in relieving pain, improving joint mobility, and enhancing quality of life in chronic inflammatory conditions such as osteoarthritis, varicose veins, and neuropathic pain [18]. The underlying mechanisms are thought to involve inhibition of platelet aggregation, regulation of vascular permeability, reduction in oxidative stress, and suppression of proinflammatory cytokine release [18,19]. Importantly, the biological effects of LS are not limited to isolated proteins; rather, it exhibits a multifaceted pharmacological profile targeting the coagulation cascade as a whole. Key components such as hirudin, bdellin, eglin, calin, and hyaluronidase contribute to fibrinolytic, anti-inflammatory, and vasodilatory effects, potentially reshaping the tumor microenvironment [15,19,20]. Moreover, inhibition of protease-activated receptor-1 (PAR-1) has been associated with the suppression of cell migration and angiogenesis, further supporting the molecular-level anticancer potential of LS [15].

In addition to experimental studies, the pharmaceutical use of leech-derived products has extended into traditional Chinese medicine, where oral preparations are clinically employed. These products are formulated as tablets or capsules and are listed among the approved medicines in the *Chinese Pharmacopoeia*. For example, Zhixiong capsules are used in the treatment of acute and chronic cerebrovascular diseases due to their ability to enhance circulation, reduce coagulation, activate meridians, and relieve stasis [21]. Shenyuandan capsules are used in the treatment of coronary heart disease and angina pectoris. They exert protective effects against myocardial ischemia/reperfusion injury, reduce oxidative stress in ischemic myocardium, and enhance myocardial antioxidant capacity. Additionally, they may attenuate atherosclerosis by promoting autophagy through epigenetic mechanisms involving DNA and Atg13 promoter demethylation. Furthermore, they have been reported to promote autophagy by inducing demethylation of the Atg13 promoter region, thereby attenuating atherosclerosis [22]. Clinically, Shenyuandan has been shown to significantly reduce procedure-related myocardial injury in patients with unstable angina compared to a placebo [23].

Taken together, these findings suggest that the anticancer, antioxidant, and anti-inflammatory effects of LS are supported not only by experimental data but also by traditional and modern clinical practices, reinforcing its potential as a valuable therapeutic agent.

The aim of this study was to investigate the biologically and pharmacologically active bioactive compounds found in crude leech saliva and to evaluate the anticancer, antioxidant and anti-inflammatory effects of crude leech saliva. For this purpose, the in vitro effects of salivary secretion obtained from *Hirudo verbana* on epithelial ovarian cancer cells were investigated. Additionally, the chemical composition of the crude leech saliva was analyzed using gas chromatography–mass spectrometry, and the potential anticancer activities of the identified compounds were further evaluated based on their interactions with molecular target proteins. This study aims to contribute to the development of novel anticancer agents from natural sources and to explore the potential of crude leech saliva as an alternative biotherapeutic agent in cancer treatment.

## 2. Materials and Methods

### 2.1. Procurement of Medicinal Leeches and Collection of Crude Leech Saliva (LS)

**Figure 1:**
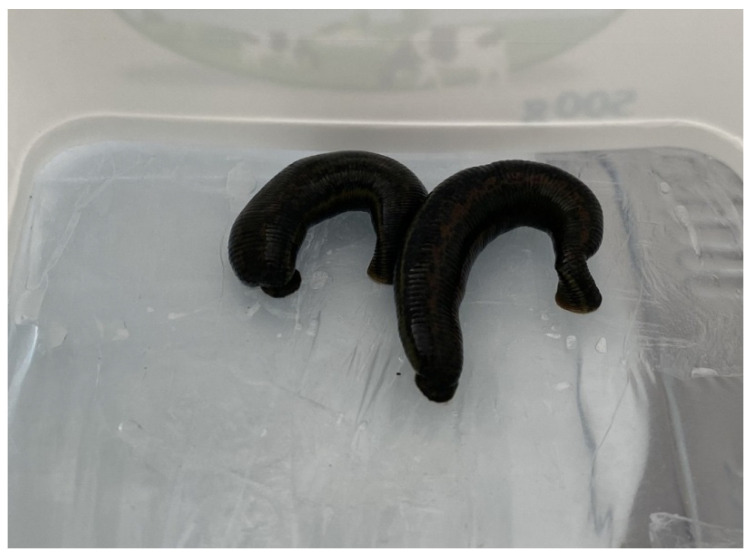
Feeding of leeches with phagostimulatory solution through parafilm membranes and collection of regurgitated LS.

Leeches were obtained from the Medicinal Leech Breeding and Research Laboratory at the Faculty of Medicine, Kırşehir Ahi Evran University (Kirşehir, Turkey), and were starved for three months to facilitate saliva collection. During this period, their water was replaced twice weekly using chlorine-free tap water. After three months, a phagostimulatory solution was prepared to feed the starved leeches. The solution was prepared by dissolving 8.766 g NaCl and 0.174 g arginine in 1 L of distilled water. The solution was heated to 37 °C using a heater and placed in a Petri dish. To mimic artificial host skin, a separate container with a base covered in parafilm was floated on the solution. Leeches were transferred into this setup to feed on the prepared solution (Figure 1). Once the leeches were fully fed, they were transferred into 15 mL sterile falcon tubes and immersed in an ice-filled container. After a 15 min waiting period, the leeches were immobilized to induce regurgitation of the solution and their saliva. Following saliva collection, the leeches were immersed in warm water (37 °C) for 15–30 min to restore their activity. The collected saliva was centrifuged at 4 °C and 2500 rpm for 10 min, and the supernatant was filtered through a 0.22 µm membrane filter. The filtrate was stored in microcentrifuge tubes at −20 °C until analysis [24]. A total of 50 *Hirudo verbana* medicinal leeches were used in this study.

### 2.2. Determination of Total Protein Content in Crude Leech Saliva (LS) by Bradford Method

The total protein content in LS was determined using the Bradford protein assay [25]. This colorimetric method is widely employed for the rapid and sensitive quantification of proteins in biological samples. In the modified version of this assay, 200 µL of Bradford reagent was added to each well of a 96-well plate. Subsequently, 20 µL of bovine serum albumin at concentrations of 500, 250, 125, 62.5, 31.25, 15.63, and 7.81 µg/mL, or 20 µL of LS diluted 1:1 with distilled water (50%, *v*:*v*), was added to the respective wells. The color change, which corresponds to the protein concentration, was measured at 595 nm using a spectrophotometer. Absorbance values for the standards and samples were uploaded into GraphPad Prism 8 software (GraphPad Software, La Jolla, CA, USA, http://www.graphpad.com, accessed on 15 December 2024) to generate a standard curve. Each sample was analyzed in triplicate, and the results were expressed as mean ± standard deviation. Since the samples were diluted 50%, the values were doubled to calculate the total protein concentration in the stock solution.

### 2.3. Chemical Characterization of Crude Leech Saliva (LS) Content by GC-MS Analysis

In this study, the chemical components of the saliva secretion obtained from *Hirudo verbana* leeches were analyzed using gas chromatography–mass spectrometry (GC-MS). The analyses were performed on an Agilent Technologies 5975 MSD/Enhanced system equipped with an HP-5MS column (30 m length, 0.25 mm internal diameter, 0.25 μm film thickness). The column’s temperature limits ranged from −0 °C to 325 °C, with a maximum usable temperature of 350 °C.

GC conditions were set as follows: the initial oven temperature was 50 °C and was increased to 310 °C in three heating ramps. The temperature program was as follows:

Ramp 1: from 50 °C to 150 °C at 20 °C/min.

Ramp 2: from 150 °C to 200 °C at 3 °C/min.

Ramp 3: from 200 °C to 310 °C at 8 °C/min (held for 10 min).

Helium was used as the carrier gas at a constant flow rate of 1.2 mL/min. Sample injection was performed in splitless mode, with 1 µL of the LS injected into the inlet heated to 250 °C. The MS source temperature was set at 230 °C and the quadrupole temperature at 150 °C. Electron ionization (EI) was employed as the ion source at an ionization energy of 70 eV. The scan range was set from 20 to 400 *m*/*z* [26].

**Figure 2:**
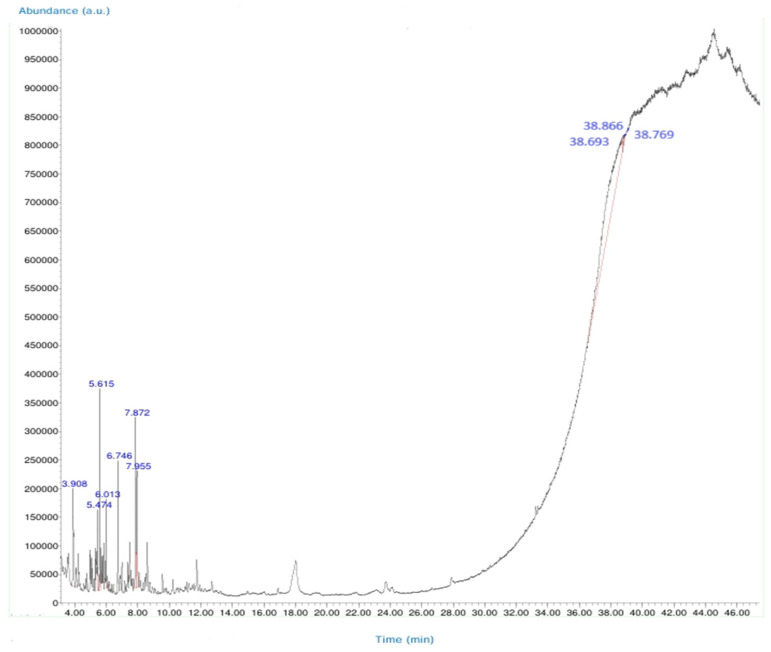
GC-MS total ion chromatogram (TIC) of bioactive compounds in LS. Each peak in the chromatogram represents the ion flux intensity and retention time of a specific compound. Among the identified bioactive compounds, GA3 (gibberellic acid) and 6-[(4-methoxyphenyl)methoxy]-4,4,5,7,8-pentamethyl-3H-chromen-2-one are notable for their biological activity. This chromatogram highlights the diversity of bioactive constituents in LS and their potential therapeutic properties.

Identified compounds were verified by comparing the obtained spectra with NIST14 and WILEY library data. Identification was confirmed based on retention times and spectral matching. GC-MS spectra and the structures of the identified compounds are presented in the corresponding figure and table (Figure 2 and Table A1).

### 2.4. Molecular Docking Analysis of Ligand–Protein Interactions

In this study, molecular docking analyses were conducted to evaluate the interaction potential between bioactive compounds identified in LS and human anti-apoptotic proteins. For this purpose, ligand–protein interactions were analyzed using both the AutoDock software (https://autodock.scripps.edu/, accessed on 27 February 2025) and the SeamDock web-based platform (https://bioserv.rpbs.univ-paris-diderot.fr/services/SeamDock/, accessed on 27 February 2025) [27,28,29].

The target proteins selected for docking were Bcl-2 and Survivin, which play key roles in the apoptosis mechanism. The three-dimensional structures of these proteins were retrieved from the Protein Data Bank (PDB) database (https://www.rcsb.org/, accessed on 27 February 2025). The PDB IDs of the selected proteins were 4MAN for Bcl-2 and 1F3H for Survivin. These crystallographic structures were used as references in the docking evaluations (Table 1).

### 2.5. Determination of Some Cytokine Levels of Crude Leech Saliva (LS)

The collected LS was thawed from −80 °C storage to room temperature and diluted to 50% (*v*/*v*) for use [17]. The results of LS were determined by doubling the obtained data. The cytokine levels were determined by ELISA (Enzyme-Linked Immuno Sorbent Assay) using TNF-α (Cat. No:E0082), IL-2 (Cat. No:E0094), and IL-6 (Cat. No:E0090) kits according to the manufacturers’ instructions. The ELISA kits were applied carefully as specified in the relevant catalogues. Each sample was analyzed in triplicate and absorbance was measured at 450 nm using a microplate reader.

### 2.6. Determination of Antioxidant Activity of Crude Leech Saliva (LS)

The antioxidant activity of LS was evaluated using the DPPH (2,2-diphenyl-1-picrylhydrazyl) radical scavenging assay, which measures the capacity of antioxidants to reduce the DPPH radical. This stable organic nitrogen radical produces a deep purple color, and the assay quantifies the reduction in this color at 517 nm. Ascorbic acid (AscA) was used as a standard at concentrations of 500, 250, 125, 62.5, 31.25, and 15.63 µg/mL. LS stock solutions were prepared at concentrations of 3.13%, 6.25%, 12.5%, 25%, and 50% (*v*/*v*) by serial dilution with distilled water. Each well of a 96-well plate received 150 µL of 1 mM DPPH solution (dissolved in ethanol) and 50 µL of either LS or standard solution. After a 30 min incubation in the dark, absorbance was measured using a microplate reader (Rayto RT-2100C, China) [30].

The percentage of remaining DPPH was calculated using the following formula:

DPPH • Percent of inhibition (%) = [A**_Control_** − A**_Sample_**/A**_Control_**] × 100

A**_Control_**: Absorbance of Control.

A**_Sample_**: Absorbance of Sample or Standard.

**Figure 3:**
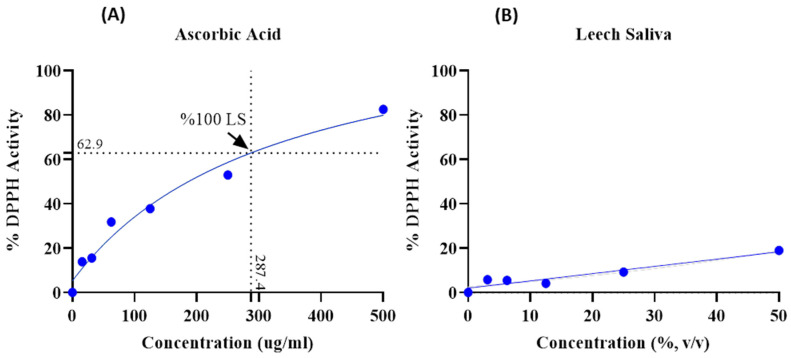
(**A**) DPPH activity level of ascorbic acid (control). (**B**) DPPH activity level of leech saliva. IC₅₀ values: ascorbic acid = 330.1 µg/mL; leech saliva > 10¹⁶%.

Each sample was analyzed in triplicate. The data obtained were graphed using the GraphPad Prism 8 program (GraphPad Software, www.graphpad.com) and IC_50_/R^2^ (Inhibitory Concentration 50/Determination Coefficient) values were determined. After evaluating all these data, the “Interpolated” feature was used in the GraphPad Prism 8 program to determine the active value of undiluted-pure LS (100% LS). In order to find the AscA equivalent of 100% LS, the data of LS were presented in the same program and obtained using the same feature (Figure 3).

### 2.7. Effect of Crude Leech Saliva (LS) on HUVEC and OVCAR3 Cell Lines

#### 2.7.1. Preparation of Cell Lines

The epithelial ovarian cancer cell line (OVCAR3, ATCC^®^ HTB-161™, Manassas, VA, USA) and the human umbilical vein endothelial cell line (HUVEC, ATCC^®^ PCS-100-010™, Manassas, VA, USA) were thawed in a sterile water bath at 37 °C. The cell culture medium was prepared using Dulbecco’s Modified Eagle Medium (DMEM, Gibco, Grand Island, NY, USA) supplemented with 10% fetal bovine serum (FBS, Sigma-Aldrich, St. Louis, MO, USA), 1% penicillin/streptomycin (Sigma-Aldrich, USA), and 2.2 g/L sodium bicarbonate. Thawed cells were transferred into sterile 25 cm^2^ cell culture flasks and incubated at 37 °C in a humidified atmosphere with 5% CO_2_. Upon reaching confluence, the cells were passaged to maintain growth [31].

To prevent any potential mycoplasma contamination, all cell lines were tested using the MycoAlert™ Mycoplasma Detection Kit (Lonza, Walkersville, MD, USA) prior to experiments, and only contamination-free cells were used in the analyses.

#### 2.7.2. Cell Counting, and Treatment with Sample

The collected cells were lysed with 1–2 mL of medium and mixed with 0.4% trypan blue at a ratio of 1:1 and used to count the cells using the Thoma chamber. The number of viable cells per mL was calculated using the following formula:

Viable cell count/mL = Average viable cell count × Dilution factor × 10^4^

The LS was filtered through a 0.22 µm membrane filter and serially diluted in sterile water at various concentrations (3.13%, 6.25%, 12.5%, 25% and 50%; *v*/*v*). After cell counting, 10^4^ cells were seeded into each well of a 96-well plate and incubated for 24 h [31]. After 24 h of incubation, 10 µL of sample was added to each well. Nothing was added to the non-treatment wells (control). After adding the sample, it was incubated for three different times (24, 48 and 72 h) and cell viabilities were determined.

#### 2.7.3. Determination of Cell Viability Using MTT Test

The effect of LS on cell viability was evaluated using the MTT (3-(4,5-dimethylthiazol-2-yl)-2,5-diphenyltetrazolium bromide) method, a widely used enzymatic method. MTT is reduced by mitochondrial succinate dehydrogenase in viable cells to form a purple, water-insoluble formazan product [32]. MTT solution was prepared at a concentration of 5 mg/mL in sterile phosphate-buffered saline.

**Figure 4:**
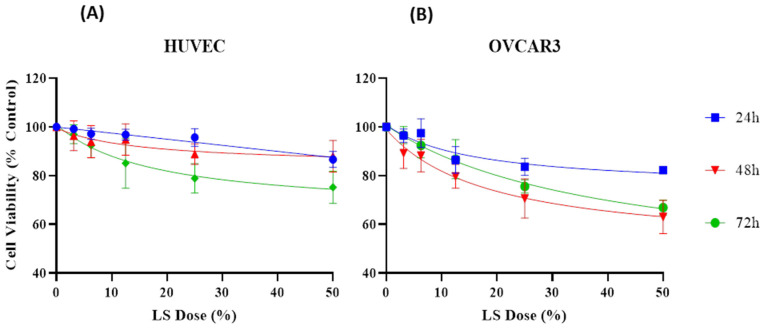
(**A**) Cell viability levels following LS treatment in HUVEC cells at 24, 48, and 72 h. (**B**) Cell viability levels following LS treatment in OVCAR3 cells at 24, 48, and 72 h.

Cells were treated with 10 µL of various concentrations of LS (3.13%, 6.25%, 12.5%, 25%, and 50%; *v*/*v*) diluted in distilled water, while the control group received no treatment. Samples were incubated with cells for 24, 48, and 72 h. After incubation, 20 µL of MTT solution was added to each well, and plates were incubated for 3 h. Subsequently, wells were aspirated, and 100 µL of dimethyl sulfoxide (DMSO) was added. Optical densities (OD) were measured at 570 nm using a microplate reader (Thermo Multiskan GO, Thermo Fisher Scientific, Waltham, MA, USA) after being shaken for 10 min in the dark. Non-treatment wells were considered 100% and the wells to which the sample was added were proportioned accordingly and each sample was repeated for at least 6 times [33] (Table 2 and Figure 4).

### 2.8. Statistical Analysis

The data from the cytokines study were analyzed using the “*Interpolate a standard curve*” feature in GraphPad Prism 8. A linear model was used for TNF-α, while “*Pade (1,1) approximant*” was applied for IL-2 and IL-6. Results were expressed as mean ± standard deviation based on triplicate analyses.

Statistical analysis of the data obtained from the MTT test results included in the study was performed using the IBM SPSS 26.0 package program. Since the number of cell cultures included in the study was 36 (n ≥ 30), the Kolmogorov–Smirnov test was applied to assess normality. As *p* < 0.05 was obtained, nonparametric analyses were subsequently performedSince the variables were *p* < 0.05, nonparametric analyses were performed. Descriptive analysis was performed for the descriptive statistics [mean (x), standard deviation (sd)] of the variables in the study, and Kruskal–Wallis H test analysis was performed for the effectiveness analysis of doses with more than two variables. Data were presented as mean ± standard deviation, and descriptive statistics for LS were summarized based on dose and time in Table 2. The IC_50_/R^2^ values were calculated using the “*[inhibitor]* vs. *response (three parameters)*” analysis in the GraphPad program.

## 3. Results

In this study, the LS was successfully isolated from the medicinal leech species *Hirudo verbana* following a three-month fasting period and feeding with a phagostimulant solution, through the regurgitation method. The obtained saliva was preserved under appropriate conditions after centrifugation and filtration steps to ensure suitability for subsequent analyses. All experimental procedures were conducted using this isolated saliva, and the detailed steps of the process are described comprehensively in the Section 2.

### 3.1. Determination of Total Protein Content of LS

In this study, the total protein content of LS was determined using the Bradford protein assay method. The reliability of the analysis was ensured by the graphical data obtained. The graph was constructed using various concentrations of bovine serum albumin (BSA) standards and showed a linear relationship (R^2^ = 0.9767). This result confirmed the sensitivity and accuracy of the method used. The protein amount of LS was calculated as 0.061 mg/mL.

### 3.2. Molecular Docking Analyses

According to the GC-MS analysis, a variety of secondary metabolites were identified in LS. Among these, compounds such as gibberellic acid (GA3) and 6-[(4-methoxyphenyl)methoxy]-4,4,5,7,8-pentamethyl-3H-chromen-2-one were selected for molecular docking analysis due to their reported biological activities. Figure 2 shows the Total Ion Chromatogram (TIC), where each peak represents a specific compound identified in LS. This chromatogram highlights the diversity of bioactive constituents present in LS and provides the chemical basis for evaluating their biological potential through in silico analysis. In this study, the molecular interactions between selected bioactive compounds identified in LS and the anti-apoptotic proteins Bcl-2 and Survivin—key regulators of programmed cell death—were evaluated. To predict ligand–protein interactions, the SeamDock web server was employed, and the results were validated and cross-checked using AutoDock software (https://autodock.scripps.edu/, accessed on 27 February 2025). As a high level of concordance was observed between the two methods, SeamDock results were used for reporting purposes.

According to the molecular docking analyses, the binding energies (ΔG) of 6-[(4-methoxyphenyl)methoxy]-4,4,5,7,8-pentamethyl-3H-chromen-2-one and gibberellic acid (GA3) with the Bcl-2 protein were calculated as −8.4 kcal/mol (3 hydrogen bonds) and −7.8 kcal/mol (2 hydrogen bonds), respectively. Similarly, these compounds exhibited binding energies of −6.6 kcal/mol (1 hydrogen bond) and −8.1 kcal/mol (4 hydrogen bonds) with the Survivin protein. These low binding energy values indicate that both molecules have the potential to form stable complexes with their target proteins. In particular, the strong binding affinity of GA3 to Survivin suggests that this compound may exert significant effects on apoptotic signaling pathways (Table 1).

Additionally, other compounds identified in LS were also subjected to docking analysis. However, these molecules exhibited relatively higher binding energies—indicating weaker binding potential—and showed less favorable interaction profiles with the anti-apoptotic proteins.

### 3.3. Determination of Some Cytokine Levels of LS

The ELISA method used to determine TNF-α, IL-2 and IL-6 cytokine levels of LS provided precise measurement of cytokine levels. It was applied as stated in the package insert of these kits and the R^2^ values of the standard curves are as follows: TNF-α = 0.9118, IL-2 = 0.9579 and IL-6 = 0.9811. Accordingly, the relevant cytokine levels of LS are as follows: TNF-α = 518.2 ng/L, IL2 = 447.4 ng/L and IL6 = 71.2 ng/L

### 3.4. Determination of the Antioxidant Activity of LS

The antioxidant activity analysis of LS revealed that both compounds had an effect on DPPH. At the maximum concentration of ascorbic acid (500 µg/mL), 82.57% activity was observed, whereas 18.92% DPPH activity was observed at the 50% concentration of LS. Ascorbic acid exhibited strong antioxidant activity at higher concentrations, while LS demonstrated limited DPPH activity. DPPH activity of undiluted-pure LS (100% LS) is 62.9% and 287.4 µg/mL AscA showed DPPH activity at this rate (Figure 3).

### 3.5. Effect of LS on Cell Viability of HUVEC and OVCAR3

According to the results obtained, in the HUVEC cell line, cell viability following treatment with 3.13% LS was determined as 99.16%, 96.36%, and 96.98% at 24, 48, and 72 h, respectively. In contrast, at the highest concentration of 50%, cell viability decreased to 86.62%, 87.96%, and 75.21%, respectively, indicating that LS exhibits limited effect toward healthy cells.

In the OVCAR3 cell line, cell viability following 3.13% LS treatment was found to be 96.44%, 89.25%, and 96.65% at 24, 48, and 72 h, respectively. However, at 50% concentration, these values declined markedly to 82.25%, 63.02%, and 66.88%, suggesting a more pronounced effect of viability of LS on cancer cells.

Due to the violation of parametric test assumptions, statistical analyses were conducted using the non-parametric Kruskal–Wallis H test. Statistically significant differences were observed among the different dose groups for both HUVEC and OVCAR3 cell lines (*p* < 0.001). Post hoc analysis revealed that, particularly in the OVCAR3 cell line, treatment with 50% LS resulted in significantly lower cell viability compared to the lower concentrations of 6.25%, 12.5%, and 3.13% (*p* = 0.000). Similarly, 25% LS also exhibited statistically significant differences when compared with 6.25% and 12.5% concentrations (Table 2 and Figure 4).

## 4. Discussion

Medicinal leech therapy (MLT), also known as hirudotherapy, is a treatment method with a significant place in both Western and Eastern medicine [34]. Historically, this practice has been used for centuries [35]; however, its systematic incorporation into modern medicine became possible following Haycraft’s 19th-century discovery of a substance that prevents blood coagulation. This compound was later identified as hirudin, a potent anticoagulant [36]. Recent studies have shown that, in addition to its anticoagulant properties, LS contains a variety of bioactive compounds with anticancer, antioxidant, and anti-inflammatory effects [24]. These multifaceted biological activities make LS a promising candidate for the development of novel therapeutic strategies derived from natural sources.

Accordingly, our study primarily conducted comprehensive analyses at both molecular and cellular levels to evaluate the anticancer effects of LS. The potential anticancer activities of the bioactive compounds identified in LS (Figure 2 and Table A1) were investigated, particularly focusing on their roles in cellular apoptotic mechanisms. In this context, molecular interactions with anti-apoptotic proteins Bcl-2 and Survivin were assessed (Table 1). GC-MS (Figure 2) and molecular docking analyses (Table 1) revealed strong binding affinities between these proteins and two key compounds—gibberellic acid (GA3) and 6-[(4-methoxyphenyl)methoxy]-4,4,5,7,8-pentamethyl-3H-chromen-2-one. According to the docking results, GA3 demonstrated the lowest binding energy with Survivin (−8.1 kcal/mol) and formed four hydrogen bonds. This suggests that GA3 and its derivatives may influence apoptotic pathways. Previous studies have shown that gibberellic acid derivatives interact with apoptotic signaling cascades and modulate cell death mechanisms [37], indicating that GA3 may act similarly by targeting anti-apoptotic proteins. Furthermore, 6-[(4-methoxyphenyl)methoxy]-4,4,5,7,8-pentamethyl-3H-chromen-2-one exhibited the strongest binding with Bcl-2 (−8.4 kcal/mol), forming three hydrogen bonds. Given that inhibition of Bcl-2 can enhance the apoptotic sensitivity of cancer cells, this compound may be considered a potential apoptotic inducer [38].

In addition, other compounds identified in LS, including 1,2-Benzisothiazol-3-amine, 2,6-Di-tert-butyl-4-methylene-2,5-cyclohexadienone, and various hydrocarbons, were also analyzed through molecular docking studies. However, these compounds exhibited relatively higher binding energy values (ranging from −3.5 to −6.3 kcal/mol), indicating weaker interactions with the anti-apoptotic proteins. In alignment with the molecular data, the selective effects of LS on cancer cells viability were also confirmed at the cellular level. Accordingly, the effects of LS were evaluated in HUVEC and OVCAR3 cells at various concentrations (3.12%, 6.25%, 12.5%, 25%, and 50%) and time points (24, 48, and 72 h) (Table 2 and Figure 4). In both cell lines, a dose-dependent and statistically significant decrease in cell viability was observed with increasing LS concentrations. This reduction was particularly pronounced in OVCAR3 cells, demonstrating the selective potential of LS against cancer cells viability.

Since the data did not meet the assumptions of parametric tests, statistical analyses were conducted using the non-parametric Kruskal–Wallis H test. Statistically significant differences were identified between dose groups in both cell lines (*p* < 0.001). Post hoc analyses revealed that in the OVCAR3 cell line, treatment with 50% LS resulted in significantly lower cell viability compared to 6.25%, 12.5%, and 3.12% concentrations (*p* = 0.000). Similarly, the 25% concentration also showed statistically significant differences when compared to 6.25% and 12.5%.

When examining the half-maximal inhibitory concentration (IC₅₀) values for cell viability, the IC₅₀ for OVCAR3 cells at 48 h was calculated as 66.14 µg/mL. In contrast, the IC₅₀ value for HUVEC cells was determined to be 283.8 µg/mL, supporting the notion that LS exerts lower toxicity on healthy cells and exhibits selective effect. These results indicate that LS may exhibit strong cell viability-reducing effects against cancer cells, especially at high concentrations. These findings are consistent with previous literature highlighting the anticancer potential of LS in cancer treatment strategies [39].

LS contains not only small bioactive chemical compounds but also various biologically active proteins, which may similarly contribute to its anticancer and anti-inflammatory effects [40]. For example, it has been demonstrated that proteins such as hirudin, found in LS, can inhibit the invasion and angiogenesis of cancer cells by suppressing the NF-κB pathway and blocking the cell cycle at the S phase, thereby reducing prostate cancer cell proliferation by up to 60%. These mechanisms suggest that LS offers anti-metastatic potential and may inhibit tumor spread through proteins such as hirustasin and bdellins [41,42,43]. Furthermore, a lyophilized leech saliva extract referred to as BPS-001 has been reported to significantly reduce metastasis in breast and prostate cancer xenograft models [44]. In alignment with previous studies demonstrating the anticancer effects of LS on the MCF-7 breast cancer cell line, the present findings further support the therapeutic potential of LS, indicating its promise as a candidate for anticancer drug development [14,44].

In addition to modulating apoptotic processes, LS has also been reported in the literature to exhibit anti-inflammatory and potential antioxidant properties [8]. In the present study, the antioxidant capacity of LS was evaluated solely using the DPPH free radical scavenging assay, and it should be noted that, by nature, the results of this assay offer limited interpretive power. In the DPPH analysis, the undiluted LS sample exhibited a lower free radical scavenging capacity compared to ascorbic acid (Figure 3).

These findings provide preliminary evidence of the antioxidant potential of LS; however, in order to make a definitive characterization of LS as an “antioxidant”, further studies incorporating complementary assays such as FRAP, ABTS, ORAC, and ferric reducing antioxidant power (FRAP) are necessary. Indeed, previous studies have emphasized the significant antioxidant effects of LS in various biological systems, often attributing this effect to lipid- and protein-based molecules [44,45]. Accordingly, LS may have a potential influence on mechanisms related to oxidative stress, and its effects could be relevant in pathological conditions such as chronic diseases and cancer. This warrants further investigation in future comprehensive studies [46,47].

Among the compounds identified in LS, gibberellic acid (GA3) is considered to contribute to the modulation of oxidative stress responses. Several studies have demonstrated that GA3 plays a role in regulating antioxidant defense mechanisms by increasing the activity of key enzymes such as superoxide dismutase (SOD), ascorbate peroxidase (APX), and glutathione reductase (GR), thereby reducing cellular oxidative damage [48]. The detection of GA3 in LS also suggests a potential link to the leech’s habitat and microbiota. GA3 is known to be synthesized by fungi such as *Fusarium fujikuroi* and *Gibberella fujikuroi*, which are commonly found in wetland ecosystems. Therefore, it should be considered that leeches may carry these fungi in their body flora or may indirectly acquire GA3 through feeding [49].

When the effects of LS on inflammation were examined, a significant decrease was observed in the levels of IL-2, IL-6, and TNF-α. This finding supports the notion that LS may exert a regulatory effect on inflammatory responses. In particular, the marked reduction in IL-6 levels suggests that LS may hold promise as a therapeutic agent for the treatment of inflammatory diseases. Previous studies have shown that natural compounds found in LS, such as quercetin and resveratrol, provide therapeutic effects by targeting inflammatory pathways [17]. Additionally, protease inhibitors such as bdellins and eglin C have been reported to suppress the excessive activity of neutral proteinases, thereby reducing tissue damage and showing potential in the treatment of inflammatory conditions like arthritis [42,43].

The findings of the present study are consistent with the existing literature on inflammation. Specifically, flavonoid-based compounds are well documented for their capacity to scavenge free radicals and regulate inflammatory responses [50]. In this context, the compound 6-[(4-methoxyphenyl)methoxy]-4,4,5,7,8-pentamethyl-3H-chromen-2-one, identified in LS, is considered to exhibit potent antioxidant and anti-inflammatory properties due to its flavonoid-like structure. The presence of hydroxyl and methoxy groups in its chemical structure is thought to enhance its radical-scavenging capacity, thereby playing a vital role in combating oxidative stress.

In summary, the findings of this study are in agreement with previous reports on the anticarcinogenic, antioxidant, and anti-inflammatory properties of LS, offering scientific support for the therapeutic potential of naturally derived bioactive compounds and serving as a valuable model for future research.

## 5. Conclusions

The findings of this study support the anti-inflammatory, antioxidant, and anticancer effects of crude leech saliva. In particular, the significant reduction observed in IL-6 levels suggests that crude leech saliva may play an effective role in modulating inflammatory responses. Furthermore, gas chromatography–mass spectrometry and molecular docking analyses revealed that certain bioactive compounds identified in crude leech saliva may interact with apoptotic pathways and exert selective effects on cancer cells. These results indicate that crude leech saliva could be considered a promising natural biotherapeutic agent for various biomedical applications, particularly in cancer research.

However, to enhance the therapeutic efficacy of crude leech saliva and support its clinical application, a more detailed analysis of its bioactive components is required. In this regard, future studies should aim to identify specific molecules within crude leech saliva, investigate their mechanisms of action on signaling pathways, and validate their therapeutic efficacy using in vivo models.

Future research should focus on exploring the effects of crude leech saliva on different cancer types and evaluating its potential clinical benefits. In addition, investigating the synergistic effects of crude leech saliva in combination with existing chemotherapeutic agents or other treatment modalities could contribute to the development of its clinical applications. In this context, comprehensive preclinical and clinical studies supporting the therapeutic activity of crude leech saliva may pave the way for the broader use of natural compounds in medical practice.

## Figures and Tables

**Table 1:** Molecular docking interactions between selected bioactive ligands with antiapoptotic proteins (Bcl-2 and Survivin).

Protein and 3D Structure	Bioactive Ligands	
	6-[(4-methoxyphenyl)methoxy]-4,4,5,7,8-pentamethyl-3H-chromen-2-one	Gibberellic acid
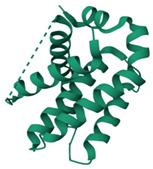 Bcl-2	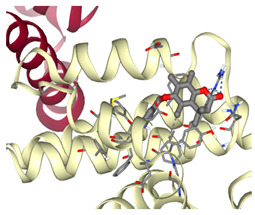 ΔG: −8.4 kcal/mol Hydrogen bonds: 3	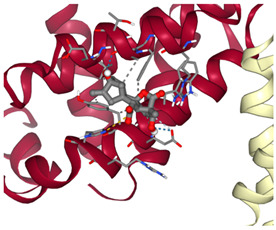 ΔG: −7.8 kcal/mol Hydrogen bonds: 2
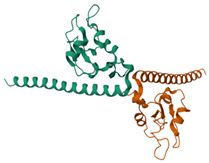 Survivin	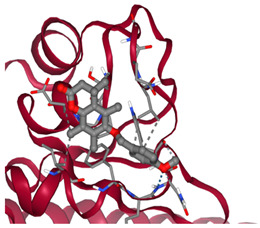 ΔG: −6.6 kcal/mol Hydrogen bonds: 1	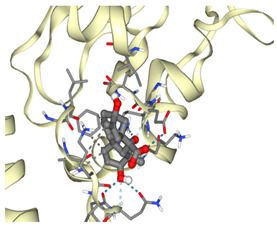 ΔG: −8.1 kcal/mol Hydrogen bonds: 4

ΔG: Binding energy values calculated by molecular docking analysis. Hydrogen bonds: Number of hydrogen bonds formed between ligand and protein.

**Table 2:** Cell viability levels following LS treatment in HUVEC and OVCAR3 cell lines at 24, 48, and 72 h.

Dose (%)	24 h (Mean ± SD)	48 h (Mean ± SD)	72 h (Mean ± SD)	24 h (Mean ± SD)	48 h (Mean ± SD)	72 h (Mean ± SD)
	HUVEC	HUVEC	HUVEC	OVCAR3	OVCAR3	OVCAR3
Control	100 ± 0	100 ± 0	100 ± 0	100 ± 0	100 ± 0	100 ± 0
3.13	99.16 ± 0.81	96.36 ± 5.58	96.98 ± 3.54	96.44 ± 2.51	89.25 ± 5.73	96.65 ± 3.20
6.25	97.20 ± 2.08	93.96 ± 6.05	92.43 ± 4.69	97.44 ± 5.35	88.22 ± 6.11	92.43 ± 4.70
12.5	96.87 ± 2.04	94.85 ± 5.93	85.08 ± 9.39	86.46 ± 5.01	79.72 ± 4.46	86.75 ± 7.32
25	95.68 ± 3.29	88.86 ± 3.85	78.88 ± 5.57	83.65 ± 3.21	70.64 ± 7.35	75.54 ± 2.52
50	86.62 ± 3.02	87.96 ± 5.91	75.21 ± 6.07	82.25 ± 1.30	63.02 ± 6.20	66.88 ± 2.80
IC_50_ R^2^	>50% 0.7582	15.14 0.3751	17.67 0.7146	15.84 0.7341	18.78 0.8228	39.77 0.8883

*p* < 0.001 according to the Kruskal–Wallis H test. Significant differences among dose groups at each time point are presented in the main text. “Very high” indicates that IC₅₀ could not be reliably determined within the tested range.

## Data Availability

Data are contained within the article.

## Appendix A

**Table A1:** GC-MS identified compounds and their 3D structures.

Molecules	Identification Information	3D Structure of Molecules
3,8-Dimethyldecane	CAS No: 17312-55-9 Retention Time: 9.995 min Peak No: 3 Area: 10,598,479 Area %: 12.08	
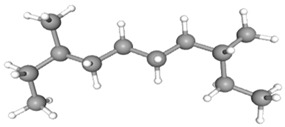
5-Methylundecane	CAS No: 1632-70-8 Retention Time: 10.906 min Peak No: 4 Area: 3,666,285 Area %: 4.18	
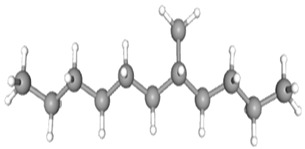
2,4-Dimethylheptane	CAS No: 2213-23-2 Retention Time: 5.473 min Peak No: 2 Area: 8,151,628 Area %: 9.29	
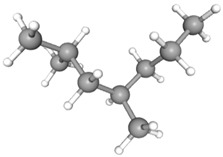
Decane	CAS No: 124-18-5 Retention Time: 8.877 min Peak No: 1 Area: 6,352,537 Area %: 7.24	
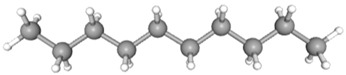
2-Methyltridecane	CAS No: 1560-96-9 Retention Time: 11.703 min Peak No: 5 Area: 3,493,446 Area %: 3.98	
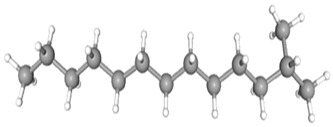
Dodecane	CAS No: 112-40-3 Retention Time: 9.393 min Peak No: 2 Area: 9,127,114 Area %: 10.40	
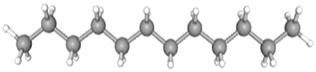
Phytane	CAS No: 638-36-8 Retention Time: 20.771 min Peak No: 6 Area: 6,319,478 Area %: 7.21	
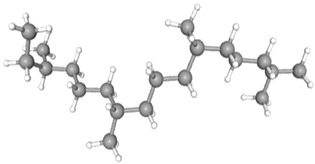
Octadecane	CAS No: 593-45-3 Retention Time: 28.013 min Peak No: 7 Area: 1,799,719 Area %: 2.05	
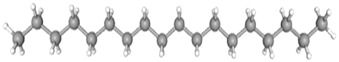
Pentadecane	CAS No: 629-62-9 Retention Time: 12.778 min Peak No: 10 Area: 4,406,601 Area %: 5.02	
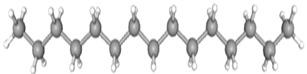
Tetradecane	CAS No: 629-59-4 Retention Time: 11.187 min Peak No: 6 Area: 3,733,756 Area %: 4.25	
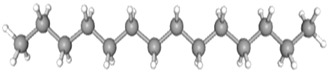
2,6-Di-tert-butyl-4-methylene-2,5-cyclohexadienone	CAS No: 2607-52-5 Retention Time: 14.682 min Peak No: 12 Area: 2,340,523 Area %: 2.67	
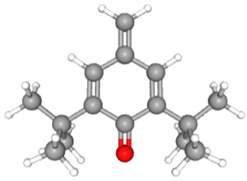
6-[(4-methoxyphenyl)methoxy]-4,4,5,7,8-pentamethyl-3H-chromen-2-one	CAS No: 39170-97-3 Retention Time: 24.063 min Peak No: 17 Area: 7,347,922 Area %: 8.36	
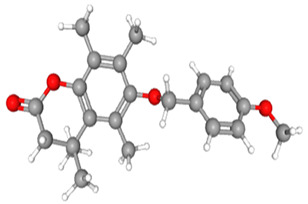
1,2-Benzisothiazol-3-amine	CAS No: 999428-58-8 Retention Time: 10.203 min Peak No: 7 Area: 1,679,551 Area %: 1.91	
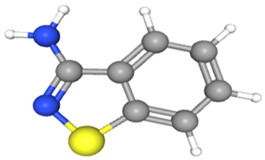
Gibberellic acid	CAS No: 77-06-5 Retention Time: 25.498 min Peak No: 18 Area: 4,691,781 Area %: 5.34	
